# Major adverse events and atrial tachycardia in Ebstein’s anomaly predicted by cardiovascular magnetic resonance

**DOI:** 10.1136/heartjnl-2017-311274

**Published:** 2017-07-06

**Authors:** Riikka Rydman, Yumi Shiina, Gerhard-Paul Diller, Koichiro Niwa, Wei Li, Hideki Uemura, Anselm Uebing, Umberto Barbero, Beatriz Bouzas, Sabine Ernst, Tom Wong, Dudley J Pennell, Michael A Gatzoulis, Sonya V Babu-Narayan

**Affiliations:** 1 NIHR Cardiovascular Biomedical Research Unit, Royal Brompton Hospital, London, England; 2 Department of Molecular Medicine and Surgery, Section of Clinical Physiology, Karolinska Institutet, Stockholm, Sweden; 3 Cardiovascular Centre, St. Luke’s International Hospital, Tokyo, Japan; 4 National Heart and Lung Institute, Imperial College, London, England; 5 Department of Cardiovascular Medicine, Division of Adult Congenital and Valvular Heart Disease, University Hospital Muenster, Muenster, Germany; 6 Città della Salute e della Scienza Hospital, University of Turin, Turin, Italy

**Keywords:** Ebstein’s anomaly, arrhythmia, sudden cardiac death, cardiovascular magnetic resonance

## Abstract

**Objectives:**

Patients with Ebstein’s anomaly of the tricuspid valve (EA) are at risk of tachyarrhythmia, congestive heart failure and sudden cardiac death. We sought to determine the value of cardiovascular magnetic resonance (CMR) for predicting these outcomes.

**Methods:**

Seventy-nine consecutive adult patients (aged 37±15 years) with unrepaired EA underwent CMR and were followed prospectively for a median 3.4 (range 0.4–10.9) years for clinical outcomes, namely major adverse cardiovascular events (MACEs: sustained ventricular tachycardia/heart failure hospital admission/cardiac transplantation/death) and first-onset atrial tachyarrhythmia (AT).

**Results:**

CMR-derived variables associated with MACE (n=6) were right ventricular (RV) or left ventricular (LV) ejection fraction (EF) (HR 2.06, 95% CI 1.168 to 3.623, p=0.012 and HR 2.35, 95% CI 1.348 to 4.082, p=0.003, respectively), LV stroke volume index (HR 2.82, 95% CI 1.212 to 7.092, p=0.028) and cardiac index (HR 1.71, 95% CI 1.002 to 1.366, p=0.037); all remained significant when tested solely for mortality. History of AT (HR 11.16, 95% CI 1.30 to 95.81, p=0.028) and New York Heart Association class >2 (HR 7.66, 95% CI 1.54 to 38.20, p=0.013) were also associated with MACE; AT preceded all but one MACE, suggesting its potential role as an early marker of adverse outcome (p=0.011).

CMR variables associated with first-onset AT (n=17; 21.5%) included RVEF (HR 1.55, 95% CI 1.103 to 2.160, p=0.011), total R/L volume index (HR 1.18, 95% CI 1.06 to 1.32, p=0.002), RV/LV end diastolic volume ratio (HR 1.55, 95% CI 1.14 to 2.10, p=0.005) and apical septal leaflet displacement/total LV septal length (HR 1.03, 95% CI 1.00 to 1.07, p=0.041); the latter two combined enhanced risk prediction (HR 6.12, 95% CI 1.67 to 22.56, p=0.007).

**Conclusion:**

CMR-derived indices carry prognostic information regarding MACE and first-onset AT among adults with unrepaired EA. CMR may be included in the periodic surveillance of these patients.

## Introduction

Mortality in Ebstein’s anomaly (EA) of the tricuspid valve relates to ventricular tachyarrhythmia, congestive heart failure and sudden cardiac death. Several predictors of adverse outcomes have been reported such as age at presentation, anatomic severity, grade of tricuspid regurgitation, cyanosis, male gender, increased cardiothoracic ratio (CTR), prolonged/fragmented QRS, reduced exercise capacity and New York Heart Association (NYHA) functional class have been reported.^1–7^ The onset of atrial tachyarrhythmia (AT) in adults is associated with significant morbidity.^8 9^ Cardiovascular magnetic resonance (CMR) is used to image adults with Ebstein’s anomaly due to unrestricted views of heart structures and its place as the gold standard for quantification of left ventricular (LV) and right ventricular (RV) volumes and function without geometrical assumption.^10–13^ Recent studies correlated CMR-derived measures in EA with known heart failure markers and/or exercise capacity,^14 15^ but its value to guide prognosis is not reported. We aimed to study the prognostic significance of CMR for significant adverse cardiac events in a large, prospective, single-centre and contemporary cohort of adult patients with unrepaired EA.

## Methods

### Patients and study design

Eighty-six consecutive patients with unrepaired EA underwent protocolised CMR and were prospectively followed for events from November 2002 until July 2014. Seven patients were lost to follow-up, thus the final study cohort consisted of 79 patients including 4 patients with prior atrial septal defect closure (surgical n=2, catheter n=2). EA was defined as apical displacement of the septal leaflet of the tricuspid valve by at least 8 mm/m^2^ body surface area in relation to the insertion of the anterior mitral valve leaflet. Patients with permanent pacemaker/automated implantable defibrillator (n=10/2) were not included in this study due to relative contraindication to CMR.

The prespecified clinical endpoint of major adverse cardiovascular events (MACEs) consisted of new-onset clinically documented sustained ventricular tachyarrhythmia/heart failure hospital admission/transplantation or death. Ventricular tachyarrhythmia was defined as ventricular tachycardia (VT) associated with presyncope/syncope, sustained VT (≥30 s) or ventricular fibrillation. Heart failure admission was defined as admission for diuresis of fluid overload not secondary to acute arrhythmia presentation. Follow-up started from CMR study and was continued until the first MACE or to surgical repair (patients were censored at surgery; n=31; 39%) or to the last clinical visit for the remainder of patients. All events during follow-up were recorded including AT for all patients, censored or not for the MACE endpoint.

A separate analysis for first-onset AT was conducted on a subset of the original study cohort excluding patients with AT prior to study inclusion, with exception for prior AT due to accessory atrioventricular pathways and Mahaim fibres (atrioventricular re-entry tachycardia (AVRT)). Only first-onset AT defined as new clinically documented sustained focal AT, atrioventricular nodal re-entry tachycardia, atrial flutter or atrial fibrillation during follow-up contributed to subsequent analysis.

Baseline data including demographics, previously documented arrhythmia, NYHA class, ECG, CTR and cardiovascular exercise testing data were obtained from medical records and clinical attendances. Mortality data from the Office for National Statistics, which registers all UK deaths, was complete for all 79 patients.

### CMR acquisition and analysis

**Figure 1:**
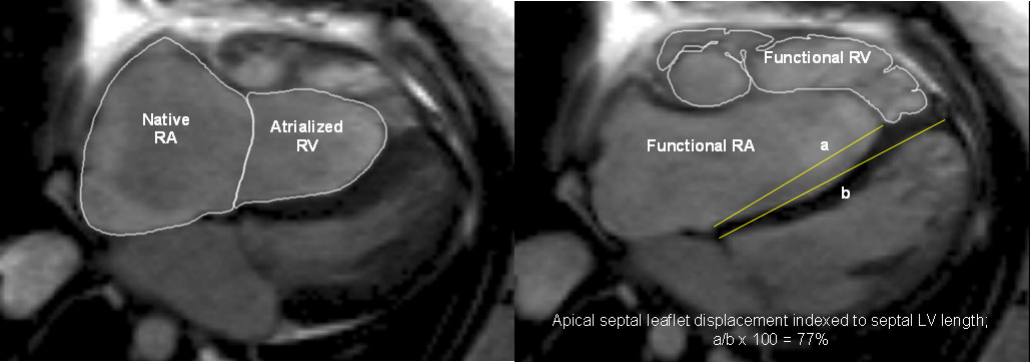
Measurements of native right atrial (RA) and atrialised right ventricular (RV) volumes, functional RV volumes and apical septal leaflet displacement/left ventricular (LV) septal length (A/B*100%). Steady-state free precession sequence and axial stack were used.

Retrospective ECG-gated balanced steady-state free precession cine images were acquired from the atrioventricular ring to the apex for measurement of LV volumes and from the diaphragm to the aortic arch for measurement of RV and atrial volumes at 1.5 T CMR. Biventricular and biatrial volumetric and functional analyses were performed by manual planimetry (CMR tools, Cardiovascular Imaging Solutions, London, UK). Native (RA) and functional right atrial and atrialised (aRV) and functional RV volumes were measured as described previously^16^ (figure 1). In short, the functional RV was defined as the aspect of the ventricle distal to the attachment points of the tricuspid valve leaflets and limited by the pulmonary valve. The malformed tricuspid valve was traced in detail to demarcate the border between the functional RV and aRV. With the aim of simple quantification of the degree of paradoxical LV motion, the magnitude of apical displacement of the septal leaflet of the tricuspid valve (defined as the distance from the atrioventricular ring to the attachment of the septal leaflet) was indexed to LV septal length measured in ventricular diastole and expressed as a percentage (figure 1). Total right/left-volume index was calculated using the equation (RA+aRV+RV)/(LA+LV) in end diastole^15^ and severity index using the equation (RA+aRV)/(RV+LA+LV).^16^ Phase contrast flow acquisitions were performed in the ascending aorta and pulmonary trunk. Cardiac output was calculated from aortic flow measurement and cardiac shunt as the ratio of pulmonary and aortic flows. Tricuspid regurgitant fraction was calculated using antegrade and retrograde flow through the pulmonary artery (PAante and PAretro) and functional RV stroke volume (RVSV) using the equation [(RVSV–PAante)/(RVSV–PAretro)] x100.^16^ LV non-compaction was defined as the end diastolic ratio of non-compacted to compacted (NC:C) myocardium >2.3:1.^17^ A single experienced observer made all measurements. Twelve random patients were remeasured by the same observer (minimum 6-month interval) as well as a second blinded observer for intraobserver and interobserver variability.

### Other investigations

Cardiopulmonary exercise testing (CPEX) was performed using a symptom-limited graded treadmill exercise within 6 months of the CMR study. Peak oxygen uptake (peak VO_2_), the per cent of predicted peak VO_2_ (peak VO_2_%), peak VO_2_ pulse, ratio of minute ventilation to carbon dioxide production (VE/VCO_2_), heart rate reserve (HRR; peak minus resting pulse rate) and anaerobic threshold were recorded. Tests were excluded from subsequent analysis if the respiratory quotient value was <1 (n=5). Venous blood was taken for brain natriuretic peptides (BNP; monoclonal antibody assay, Shionoria, Schering, West Sussex, England) at the time of the CMR examination. CPEX and BNP were performed as a part of the clinical care and were therefore not available for all patients. NYHA classification and QRS duration/QRS fractionation from standard 12-lead ECG were collected at the time point of the CMR exam. Chest X-ray within 1 month of the CMR study was included for measurement of CTR.

### Statistical methods

Continuous data are presented as mean (±SD) or median (first and third quartiles) as appropriate. Comparisons between groups were made using t-test, Mann-Whitney test and Fisher’s exact test as appropriate. Correlation was tested with Pearson’s coefficient or Spearman’s r. The association between variables and event-free survival was tested using a Cox proportional hazards model and survival curves were constructed to illustrate the impact of impaired ventricular function, RV to LV volume ratio and apical septal leaflet displacement/LV septal length based on the results of the analyses. The proportional hazards assumption was verified using generalised linear regression analysis, testing for a non-zero slope of the scaled Schoenfeld residuals in addition to visual inspection of the graphs of the regression. Due to the relatively small number of outcome events, we focused on univariable analyses. Variability was expressed as the mean per cent error, derived as the absolute difference between two sets of observations, divided by the mean of the two sets of observations. All tests were two-sided and a p value of <0.05 was considered significant. Analysis was performed using SPSS V.22.

## Results

### Patient characteristics

Seventy-nine patients with EA (mean age 37.1±15.1 years, 36 males) were prospectively followed for a median 3.4 (range 0.4–10.9) years (table 1). Mean systolic and diastolic blood pressures were 118±16 mm Hg and 77±11 mm Hg, respectively. Resting oxygen saturation was ≤90% in 5% and plasma BNP levels were increased in 76% (>5 pmol/L). The QRS duration was prolonged in 14% (>110 ms). In total, 45 patients (57%) had severe and 23 (29%) moderate tricuspid regurgitation, respectively. Eighty-six per cent of patients (68/79) had increased functional RV end diastolic volume index (>100 mL/m^2^) clearly above the range for normal RV,^18^ whereas cardiac index was impaired in 16% (<2.3 L/min/m^2^).^19^ No significant difference was found in the tested variables presented in table 1 between patients with data available from CPEX and BNP (n=50 for both) compared with patients without these data. No difference in left ventricular ejection fraction (LVEF) or stroke volume, or right ventricular ejection fraction (RVEF) was seen when patients with LV non-compaction (n=15) were compared with the reminder. LVEF correlated with RVEF (r=0.58, p<0.001) and inversely with NYHA class, aRV indexed volume (r_s_=−0.37, p=0.001 for both) and functional RV end systolic volume (r_s_=−0.36, p=0.001). RVEF and LV stroke volume both related inversely with NYHA class (r_s_=−0.38, p=0.001 and r_s_=−0.44, p<0.001, respectively). All cardiac events during follow-up are presented in table 2. Patients referred for surgery on clinical grounds during follow-up (n=31; 39%) differed significantly from those without surgery (table 1). Post surgery there was no difference in RVEF (45.0±5.7 vs 44.3±8.0, p=0.743), but LVEF and peak VO_2_ ameliorated (52.2±6.2 vs 65.7±8.9, p<0.001 and 19.5±6.0 vs 22.8±5.3, p=0.003, respectively).

**Table 1:** Patient characteristics for the whole cohort, and for patients with surgery versus patients without surgery during the follow-up

	All patients (n=79)	Patients with surgery* (n=31)	Patients without surgery (n=48)	p Value†
Clinical status				
Age at CMR, years	37.1±15.1	35.1±15.5	38.3±15.0	0.361
Male gender, n (%)	36 (46)	13 (42)	23 (48)	0.602
Body surface area, m^2^	1.68±0.12	1.68±0.12	1.69±0.12	0.630
NYHA functional class 1/2/3, n (%)	30 (38)/39 (49)/10 (13)	4 (13)/24 (77)/3 (10)	26 (54)/15 (31)/7 (15)	**<0.001**
Atrial septal defect/patent foramen ovale, n (%)	25 (32)/24 (30)	15 (48)/12 (39)	10 (21)/12 (25)	**<0.001**
Sinus rhythm, n (%)‡	73 (92)	29 (94)	40 (83)	0.363
QRS duration, ms	130.4±26.1	140.9±24.5	120.5±24.0	**0.002**
QRS fractionation, n (%)	32 (49)	23 (72)	9 (27)	**<0.001**
Accessory pathway, n (%)	12 (15)	4 (13)	8 (17)	**0.649**
Oxygen saturations at rest in room air (%)	97 (96–98)	96 (94–98)	98 (96–98)	**0.023**
Previously documented arrhythmia, n (%)	19 (24)	7 (23)	12 (25)	0.806
AVRT (WPW)/AT/paroxysmal AF, n (%)	11 (14)/5 (6)/3 (4)	3 (10)/4 (13)/0 (0)	8 (17)/1 (2)/3 (6)	–
LV non-compaction, n (%)§	15 (19)	12 (39)	3 (6)	0.090
Cardiothoracic ratio (n=71)	0.57±0.08	0.58±0.04	0.56±0.09	0.463
Brain natriuretic peptide, pmol/L (n=50)	10.5 (5.8–20.8)	15.0 (8.0–27.0)	7.0 (5.0–15.0)	**0.017**
Peak VO_2_, mL/kg/min (n=50)	22.0±7.5	19.5±6.0	24.0±8.0	**0.032**
Peak VO_2_% (n=50)	67.3±22.1	58.7±17.2	74.0±23.5	**0.014**
CMR				
Qp:Qs	1.0±0.2	1.0±0.2	1.1±0.3	0.579
Cardiac index (L/min/m^2^)	3.3±1.3	2.9±0.9	3.6±1.5	**0.021**
Functional RA volume index, mL/m^2^	147.5 (104.7–214.5)	211.8 (173.7–246.3)	123.1 (98.8–160.0)	**0.001**
Native RA volume index, mL/m^2^	112.5 (83.6–172.6)	160.3 (114.7–185.1)	100.5 (81.1–133.3)	**<0.001**
Atrialised RV volume index, mL/m^2^	40.7 (25.8–69.3)	63.0 (45.4–92.5)	33.5 (25.1–53.1)	**0.001**
Tricuspid regurgitant fraction, %	33.7 (24–56)	51.8 (30–61)	26.3 (23–36)	**0.004**
Apical septal leaflet displacement, mm	46.6±15.4	53.0±17.3	42.9±12.8	**0.004**
Apical septal leaflet displacement indexed, %	53.5±18.6	60.0±19.9	49.4±16.8	**0.013**
Functional RV end diastolic volume index, mL/m^2^	120.2 (92.5–162.7)	152.9 (120.0–189.1)	108.7 (86.0–126.7)	**<0.001**
Functional RV end systolic volume index, mL/m^2^	63.9 (46.2–87.5)	86.9 (65.0–105.1)	51.6 (41.1–66.3)	**<0.001**
Functional RV stroke volume index, mL/m^2^	57.4 (46.0–78.0)	74.2 (55.6–85.3)	50.2 (41.9–65.9)	**<0.001**
Functional RV ejection fraction, %	46.5±7.1	45.0±5.7	47.4±7.8	0.144
Functional RV/LV end diastolic indexed volume ratio	1.58 (1.21–2.32)	2.32 (1.72–2.90)	1.34 (1.06–1.71)	**<0.001**
LV end diastolic volume index, mL/m^2^	81.8 (67.5–93.6)	72.8 (65.2–83.7)	85.7 (73.9–101.9)	**0.004**
LV end systolic volume index, mL/m^2^	38.1 (31.3–45.5)	35.4 (26.5–43.3)	39.9 (31.8–48.4)	0.117
LV stroke volume index, mL/m^2^	41.2 (35.2–50.9)	38.8 (32.9–46.9)	44.5 (36.2–53.9)	**0.014**
LV ejection fraction, %	53.6±7.4	52.2±6.2	54.5±8.0	0.392
Total R/L volume index	2.7 (1.9–4.4)	4.4 (2.9–6.0)	2.1 (1.8–3.2)	**<0.001**
Severity index	0.70 (0.46–0.92)	0.89 (0.70–1.01)	0.62 (0.42–0.83)	**0.010**

*Tricuspid valve replacement (bioprosthesis/mechanic n=20/1), tricuspid valve repair (n=11), RV/RA plication (n=20/16), Patent Foramen Ovale(PFO)/Atrial Septal Defect (ASD) closure (n=10/12), ASD enlargement (n=1), MACE/cryoablation (n=5/13). Selection for surgery was based on clinical symptoms including exercise intolerance.

†Mann-Whitney/t-test/χ^2^.

‡Sinus rhythm in 73 and established AF in 6.

§Non-compacted to compacted myocardium (NC/C) ratio >2.3.

AF, atrial fibrillation; AT, atrial tachycardia; AVRT, atrioventricular re-entry tachycardia; CMR, cardiovascular magnetic resonance; LV, left ventricular; MACE, major adverse cardiovascular event; NYHA, New York Heart Association; Qp:Qs, ratio of pulmonary to aortic flow; RA, right atrium; RV, right ventricular; WPW, Wolff-Parkinson-White syndrome

Statistically significant p values are displayed in bold format.

**Table 2:** Clinical outcomes of unrepaired Ebstein’s anomaly patients at median 3.4 (range 0.4–10.9) years follow-up

All cardiac events during follow-up	Patients (n=79)
Death (sudden in all), n (%)*	4 (5%)
Cardiac transplantation	1
Congestive heart failure, n (%)†	3 (4%)
New-onset VT after CMR, n (%)	12 (15%)
Sustained VT, n	3
Non-sustained VT, n	9
First-onset atrial tachyarrhythmia after CMR, n (%)	17 (22%)
Atrial tachycardia	10
Atrial fibrillation	7

Patients experiencing death/sustained VT/heart failure/cardiac transplantation; NYHA class ≥3 n=3, chronic atrial fibrillation n=2, accessory pathway n=3, QRS duration ≥120 ms n=5, exercise intolerance n=2, decreased right ventricular ejection fraction n=6, decreased left ventricular ejection fraction n=4.

Following a MACE, three patients went on for tricuspid valve surgery, one for cardiac transplantation.

*Two confirmed cardiac deaths, two unknown causes but sudden.

†One patient required hospital admission for diuresis and two patients deteriorated to NYHA class 4.

CMR, cardiovascular magnetic resonance; MACE, major adverse cardiovascular event; NYHA, New York Heart Association; VT, ventricular tachycardia.

### Association of CMR-derived variables with MACE

**Figure 2:**
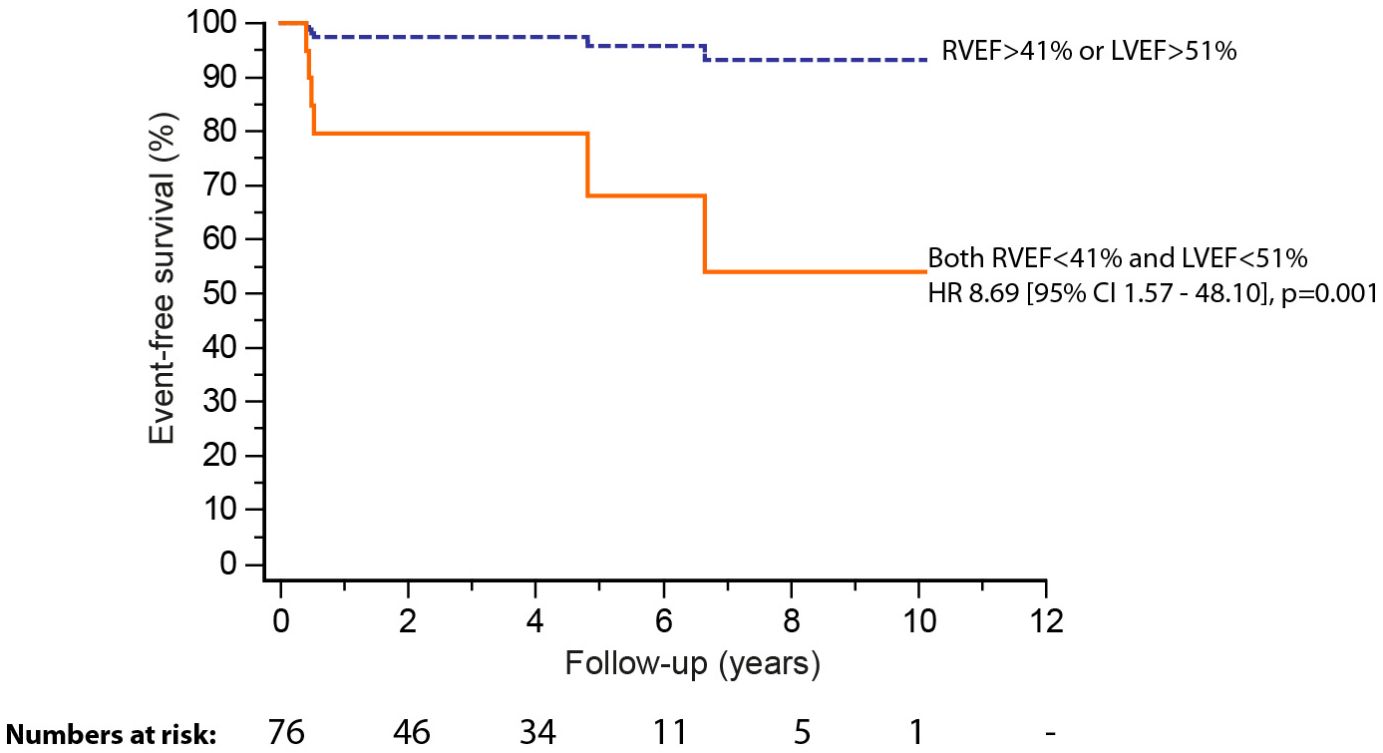
Freedom from death, sustained ventricular tachycardia, heart failure hospital admission or cardiac transplant stratified by lower quartile ejection fraction (left ventricular ejection fraction (LVEF) <51%, right ventricular ejection fraction (RVEF) <41%).

At latest follow-up, six (7.6%) patients had reached the composite endpoint (median time to event of 3.4 years). Events contributing to the composite endpoint were three sustained VT and three sudden deaths; one patient that presented with VT was later hospitalised for heart failure and died 2 years after heart transplantation (table 2). MACEs were preceded by clinically documented AT in all but one patient (p=0.011). Univariable predictors of MACE are summarised in table 3. Survival analysis showed an almost ninefold higher rate of MACE during follow-up in patients with biventricular impairment compared with RV or LV impairment only (lower quartile RVEF; <41% and LVEF; <51%; HR 8.69, 95% CI 1.57 to 48.10, p=0.001) (figure 2). RVEF and LVEF were significantly related (r=0.58, p<0.001). When tested solely for mortality, all univariable predictors with the exception of previous documented AT remained significant. The presence of LV non-compaction was not associated with outcome. No MACE occurred in patients post surgery.

**Table 3:** Association of clinical features, cardiopulmonary exercise and CMR with MACE (ventricular tachycardia/heart failure/transplant/death) in unrepaired Ebstein’s anomaly patients during 3.5±2.6 years follow-up

Patient factors (n=79)	Per*	HR	95% CI	p Value†
Clinical features				
Age at inclusion	↑1 year	0.964	0.908 to 1.024	0.236
Male gender	–	0.407	0.074 to 2.228	0.300
NYHA class>2	–	***7.659***	***1.535 to38.204***	***0.013***
Previous atrial tachyarrhythmia	–	***11.155***	***1.299 to95.813***	***0.028***
Atrial septal defect/patent foramen ovale	–	1.279	0.233 to 7.004	0.777
QRS duration	ms	1.016	0.980 to 1.054	0.393
QRS fractionation	–	1.000	0.063 to 15.988	1.000
Accessory pathway	–	0.236	0.043 to 1.297	0.097
Oxygen saturation at rest	↓1%	1.129	0.891 to 1.431	0.314
Brain natriuretic peptide (n=50)	↑1 pmol/L	0.981	0.841 to 1.144	0.807
Cardiothoracic ratio>65	–	2.182	0.227 to 20.985	0.499
Cardiopulmonary exercise capacity (n=50)				
Heart rate reserve	↓1 bpm	1.033	0.971 to 1.189	0.302
Per cent predicted VO_2_	↓1%	1.081	0.978 to 1.193	0.128
VE/VCO_2_ slope	–	0.953	0.837 to 1.083	0.459
Right heart CMR measures				
Functional right atrial indexed volume	↑5 mL/m^2^	1.009	0.983 to 1.037	0.493
Native right atrial indexed volume	↑5 mL/m^2^	1.008	0.979 to 1.038	0.587
Atrialised RV indexed volume	↑5 mL/m^2^	1.071	0.953 to 1.203	0.252
Tricuspid regurgitant fraction	↑1%	0.998	0.949 to 1.050	0.953
Apical septal leaflet displacement	↑1 mm	1.042	0.984 to 1.104	0.160
Apical septal leaflet displacement/LV septal length	↑1%	1.040	0.989 to 1.093	0.124
Functional RV end diastolic volume index	↑5 mL/m^2^	1.021	0.989 to 1.093	0.427
Functional RV stroke volume index	↓10 mL/m^2^	1.018	0.845 to 1.280	0.904
Functional RV ejection fraction	↓5%	*2.058*	***1.168* to *3.623***	*0.012*
Left heart CMR measures				
LV end diastolic volume index	↑5 mL/m^2^	0.925	0.744 to 1.149	0.481
LV stroke volume index	↓10 mL/m^2^	*2.817*	***1.121 to7.092***	*0.028*
LV ejection fraction	↓5%	*2.347*	***1.348 to4.082***	*0.003*
Cardiac index	↓100 mL/min/m^2^	*1.171*	***1.002 to1.366***	*0.047*
Combined right and left heart CMR measures				
Functional RV/LV end diastolic indexed volume ratio	↑1 unit	1.178	0.645 to 2.150	0.594
Total right/left volume index	↑1 unit	1.059	0.807 to 1.390	0.678
Severity index volume	↑1 unit	1.402	0.388 to 5.056	0.606

*Unit change in the parameter tested for hazard analysis is based on clinical relevance.

†p Values are derived from univariable Cox proportional hazard analysis.

CMR, cardiovascular magnetic resonance; LV, left ventricular; MACE, major adverse cardiovascular event; NYHA, New York Heart Association; RV, right ventricular.

Significant univariable predictors of MACE are formatted bold and italic.

### Association of CMR-derived variables with first-onset AT

**Figure 3:**
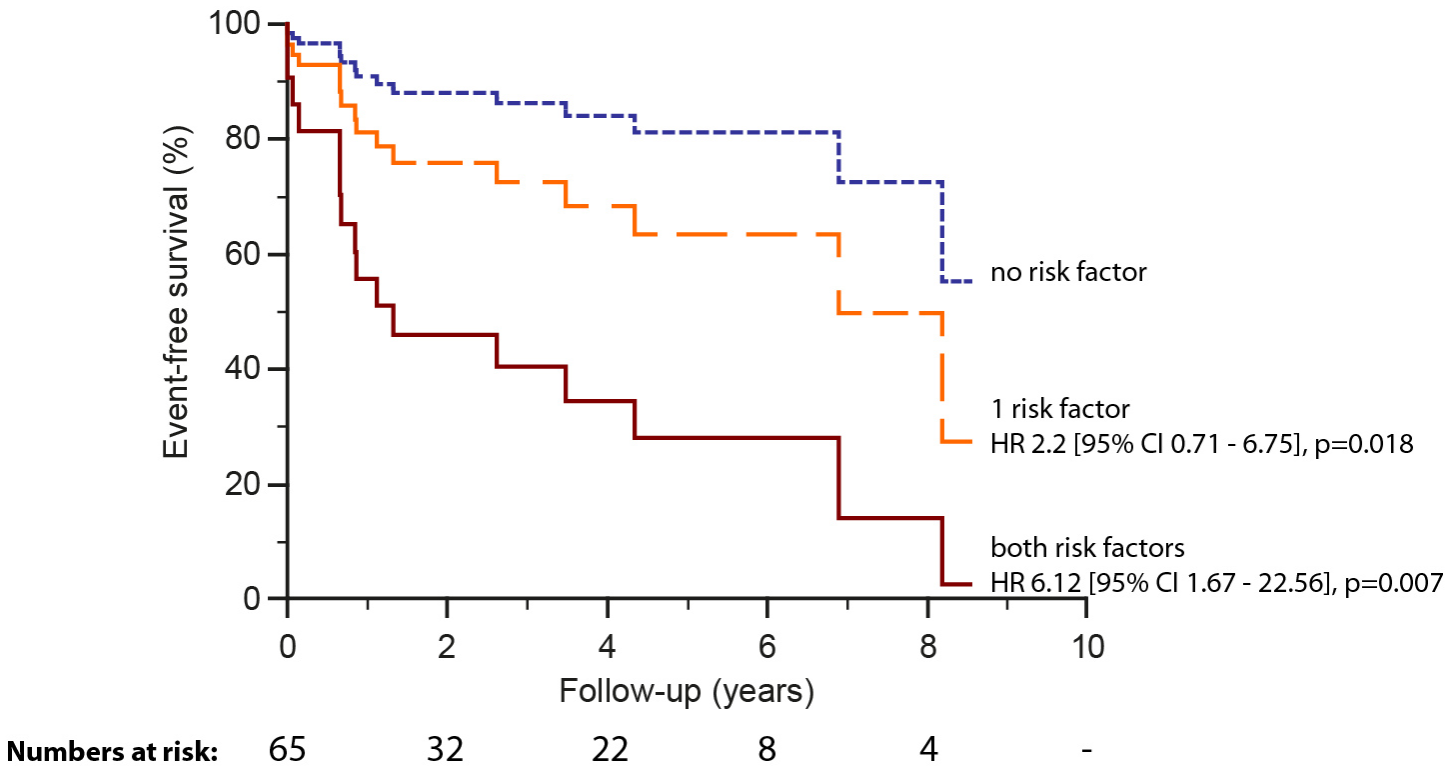
Survival curves for first-onset atrial tachycardia (n=65) stratified by univariable predictors with upper quartile functional right ventricular:left ventricular (LV) ratio (>2.4) and apical septal leaflet displacement/LV septal length (>67%) cut-offs accordingly.

Sixty-five patients (mean age 34.5±14.8 years, 25 males) formed a subset from the original study cohort for analysis of first-onset AT (n=17). Cumulative mid-term freedom from first-onset AT at 6 months, 1 year, 5 years and 7 years was 93.7%, 83.6%, 70.3% and 60.3%, respectively. Univariable predictors are summarised in table 4. Per cent predicted peak VO_2_ was borderline significant (p=0.052). As the number of outcome events was relatively small we did not perform multivariable analysis. Instead, we sought to estimate the combined prognostic value of the univariable predictors. Therefore, several models were established including subsets of the univariable variables. When upper quartile RV/LV end diastolic ratio (>2.4) and upper quartile apical tricuspid septal leaflet displacement indexed to total LV septal length (>67%) were combined, the created model yielded higher rate of first-onset AT (HR 6.12, 95% CI 1.67 to 22.56, p=0.007) compared with other combined models or models using a single univariable parameter (figure 3).

**Table 4:** Association of CMR with first-onset atrial arrhythmia during follow-up

Patient factors (n=65)	Per*	HR	95% CI	p Value†
Right heart CMR measures				
Functional right atrial indexed volume	↑5 mL/m^2^	***1.026***	***1.008 to1.044***	*0.005*
Native right atrial indexed volume	↑5 mL/m^2^	***1.026***	*****1.008 to1.044*****	*0.005*
Atrialised RV indexed volume	↑5 mL/m^2^	1.046	0.967 to 1.132	0.262
Tricuspid regurgitant fraction	↑1%	1.015	0.986 to 1.044	0.314
Apical septal leaflet displacement	↑1 mm	1.035	0.992 to 1.081	0.112
Apical septal leaflet displacement/LV septal length	↑1%	***1.034***	******1.001* to *1.068******	***0.041***
Functional RV end diastolic volume	↑5 mL/m^2^	***1.046***	******1.004 to1.089******	***0.033***
Functional RV stroke volume index	↓10 mL/m^2^	0.923	0.769 to 1.109	0.394
Functional RV ejection fraction	↓5%	***1.543***	******1.103 to2.160******	***0.011***
Left heart CMR measures				
LV end diastolic volume index	↑5 mL/m^2^	0.926	0.813 to 1.056	0.253
LV stroke volume index	↓10 mL/m^2^	1.346	0.857 to 2.110	0.197
LV ejection fraction	↓5%	1.040	0.951 to 1.136	0.395
Cardiac index	↓100 mL/min/m^2^	1.001	0.997 to 1.005	0.594
Combined right and left CMR heart measures				
Functional RV/LV end diastolic indexed volume ratio	↑1 unit	***1.546***	******1.140 to2.097******	***0.005***
Total right/left volume index	↑1 unit	***1.183***	******1.063 to1.317******	***0.002***
Severity Index volume	↑1 unit	2.626	0.672 to 10.259	0.165

*Unit change in the parameter tested for hazard analysis is based on clinical relevance.

†p Values are derived from univariable Cox proportional hazard analysis.

CMR, cardiovascular magnetic resonance; LV, left ventricular; RV, right ventricular.

Significant univariable predictors of first-onset sustained atrial arrhythmia are formatted bold and italic.

### Reproducibility of CMR measurements

The coefficient of variability for intraobserver reproducibility for the native RA/aRV/functional RV end diastolic volume/end systolic volume/stroke volume/EF was 2.3%/2.1%/1.4%/2.4%/1.7%/2.4%, respectively. Interobserver variabilities were 3.4%/3.2%/3.0%/4.5%/3.3%/4.0%, respectively.

## Discussion

In this prospective study of adults with unrepaired EA, CMR-derived markers of biventricular function, namely RV and LV systolic dysfunction, reduced LV stroke volume and reduced cardiac index, were associated with mortality and sustained VT. Second, AT was common, preceded VT and death in all but one patient, and was associated with right but not left-sided impairment. First onset of AT showed strongest correlation with a composite of ventricular volumes and displacement of the septal tricuspid valve leaflet indexed to LV length. Indexing to LV length incorporates a measure of paradoxical interventricular septal motion and consequent impaired LV filling. Lastly, our data suggest that RV impairment precedes biventricular involvement which precedes mortality.

### Association of CMR-derived biventricular EF and cardiac output with MACE

We found that standard CMR-derived parameters for ventricular dysfunction were associated with mortality and sustained VT. A 5% decrease in RVEF and LVEF, respectively, was associated with a twofold higher rate of MACE over a median of 3.4 years follow-up. In addition, biventricular impairment (lower quartile RVEF <41% and LVEF <51%, respectively) carried nearly ninefold increased rate of adverse outcome. LV stroke volume (from manual planimetry) was related to cardiac index (from flow mapping) as might be expected and like RVEF or LVEF also was associated with onset of MACE. A 10 mL/m^2^ decrease in LV stroke volume increased the probability of MACE nearly threefold.

RVEF and LVEF were significantly inter-related providing evidence of ventricular–ventricular interaction in unrepaired patients. This can be explained by impaired systolic RV contractility combined with tricuspid regurgitation and loss of synchrony between the native RA and aRV resulting in decreased effective RVSV and hence reduced LV preload and stroke volume. Right-sided dilatation, in the presence of an intact pericardium, causes undesirable changes to LV size and geometry, ‘stiffer’ LV and impaired LV diastolic filling. Right-sided volume overload additionally causes leftward bulging of the interventricular septum, leading to limited filling and discoordinate contraction of the LV.^9 20–25^

Previous studies have reported several parameters as predictors of outcomes in EA.^1–5^ When tested in the present study, only NYHA class >2 proved to be prognostic. We could not confirm the previous finding of peak VO_2_% and HRR as predictors of outcomes. However, the previous study^2^ included a more heterogeneous patient sample with patients that had already undergone tricuspid valve surgery and the endpoints included elective tricuspid valve surgery (16 out of 22 endpoint events); clinical decision to refer for surgery was made unblinded to peak VO_2_%.^2^ In the present study, peak VO_2_% was lower in patients referred for tricuspid valve surgery (n=31) during follow-up compared with the remainder, as expected.^26^ Most of the studied variables were more adversely affected in the group that was selected for surgery compared with the rest. This might suggest risk prediction of MACE is enhanced using the CMR-derived combined variable of lower quartile RVEF <41% and LVEF <51% and this could be tested for a potential role in facilitating earlier and more accurate surgical decision-making. However, rather than using a single variable for decision-making we suggest for clinical purposes a combination of the clinical features, NYHA class and arrhythmia propensity, with imaging markers.

### Association of CMR with AT; an early marker of MACE

We showed that AT was associated with sustained VT and death during follow-up, suggesting its potential role as an early marker of adverse outcome. Furthermore, AT occurred mostly in the presence of deranged right-sided CMR indices, suggesting that right-sided dilatation and dysfunction occur first and may influence the later development of left-sided pathology. Different mechanisms have been suggested for the functional RV dilatation and dysfunction we observed in the present study including volume overload due to tricuspid regurgitation, thinner free wall of the dilated functional RV with fewer myocardial fibres and disrupted myofibril continuity. The enlarged RA, compensatory to the chronic haemodynamic stress due to right-sided volume overload and functional RV stiffness/systolic impairment, may become arrhythmogenic.^27–30^ We propose that the magnitude of displacement of the septal tricuspid valve leaflet indexed to total LV septal length reflects not only the severity of the atrialisation of the RV, but more specifically the degree of the paradoxical interventricular septal motion leading to adverse changes in LV geometry and impaired LV filling.^9 20–25^ In addition, parameters recently proposed for assessing disease severity due to their relationship to known heart failure parameters, such as the functional RV/LV end diastolic volume ratio and total right/left volume index, also correlated with outcomes in our cohort.^14 15^

As the number of univariable predictors was high, we sought to ascertain the discriminative value of common and easily measured CMR parameters and found the combination of two such measures resulted in a greater predictive value than when used alone. Our findings revealed a sixfold increased rate for first-onset AT when a model including functional RV/LV end diastolic volume ratio and apical tricuspid septal leaflet displacement indexed to total LV septal length was used (upper quartile RV/LV >2.4 and displacement index >67%, respectively). The functional RV/LV end diastolic volume ratio reflects the inter-related change in the ventricular volumes within an intact pericardium (transversal interaction). Combining the RV/LV end diastolic volume ratio with the indexed tricuspid septal leaflet displacement adds longitudinal interaction (atrium and ventricle) in the model. We could not confirm an association between tricuspid regurgitation and outcome, perhaps due to the high prevalence of tricuspid regurgitation (moderate/severe in 80%).

### Limitations

Patients with permanent pacemaker/automated cardiac defibrillator were not included which may cause selection bias. As the CPEX and BNP were performed as a part of the clinical care, there is a limitation in testing prospective CMR data against CPEX and BNP. Nevertheless, we found no significant difference in the tested variables presented in table 1 between patients with data available from CPEX and BNP compared with patients without these data. RA and RV volumes and function are more time consuming and more challenging to measure in EA compared with structurally normal RV and consensus on the best method for quantification is lacking. We performed manual planimetry and opted to use the gold standard methods for each cardiac chamber for best interobserver and intraobserver reproducibility which we found to be satisfactory. All measures were made by a single observer measuring anonymised scans. Due to the relatively small number of outcome events, we could not perform multivariable analysis and therefore cannot determine which of the parameters being significant in univariable analyses would remain significant in multivariable analyses. The CMR-derived indices we found to be associated with outcomes in our cohort should be examined in future, larger studies with a longer period of observation to ascertain any role towards optimal timing of surgery, determine if they are modifiable by treatment such as surgery and whether their modification improves prognosis.

## Conclusions

CMR-derived biventricular impairment and diminished LV stroke volume showed strong association with mortality and VT in a large, contemporary cohort of adults with unrepaired EA. AT preceded VT and death. First onset of AT was best predicted by a composite of ventricular volumes and displacement of the septal tricuspid valve leaflet indexed to LV length. These preliminary data support incorporating CMR as a prognostic tool in the periodic assessment of patients with EA.

**Key messages**

**What is already known on this subject?**

Patients with Ebstein’s anomaly (EA) are at risk of tachyarrhythmia, congestive heart failure and sudden cardiac death. Recent studies correlated cardiovascular magnetic resonance (CMR)-derived measures in EA with known heart failure markers and/or exercise capacity, but its value to guide prognosis is not reported.

**What might this study add?**

We studied the prognostic value of CMR for significant major adverse cardiac events (MACEs) in a large, prospective, single-centre and contemporary cohort of unrepaired adult patients. CMR-derived markers of biventricular function were associated with mortality and sustained ventricular tachycardia (VT). Atrial tachyarrhythmia (AT) was common, preceded VT and death, and was associated with right-sided impairment. First onset of AT showed strongest correlation with a composite of ventricular volumes and displacement of the septal valve leaflet/LV length. Right ventricular impairment precedes biventricular involvement which precedes mortality.

**How might this impact on clinical practice?**

Risk prediction of MACE is enhanced using the CMR-derived biventricular impairment and this could be tested for a potential role in facilitating earlier/more accurate surgical decision-making. These data support incorporating CMR as a prognostic tool in the periodic assessment of patients with EA. However, rather than using a single variable for decision-making we suggest for clinical purposes a combination of the clinical features/New York Heart Association class/arrhythmia propensity, with imaging markers.
